# Bacillus Pertussis

**Published:** 1913-11

**Authors:** 


Bacillus Pertussis of Bordet and Gengou has been subjected to the demands of Koch's laws by Mallory and Horner and apparently may be accepted as the specific cause. *Jour. of Med. Research,* Vol. 27, page 391, 1913. The organisms are massed together and interfere with the action of the cilia of the trachea and bronchi.

